# Exploring the neuroprotective mechanism of Tongqiao Huashuan granules in vascular dementia based on PLCγ1/IP3R signaling pathway

**DOI:** 10.3389/fnagi.2026.1719664

**Published:** 2026-03-04

**Authors:** Xianhui Huang, Jiajia Liu, Yanna Ren, Dingding Liu, Jing Cai, Liping Cao, Tong Lei, Yuanhua Wu

**Affiliations:** 1 First Clinical Medical College, Guizhou University of Traditional Chinese Medicine, Guiyang, China; 2 School of Humanities and Management, Guizhou University of Traditional Chinese Medicine, Guiyang, China; 3 School of Pharmaceutical Sciences, Guizhou University of Traditional Chinese Medicine, Guiyang, China; 4 Department of Neurology, The First Affiliated Hospital of Guizhou University of Traditional Chinese Medicine, Guiyang, China; 5 Institute of Basic Theory for Chinese Medicine, China Academy of Chinese Medical Sciences, Beijing, China

**Keywords:** intracellular calcium overload, oxidative stress, PLCγ1/IP3R signaling pathway, TQHS granules, vascular dementia

## Abstract

**Introduction:**

Vascular dementia (VaD) is closely associated with oxidative stress and intracellular calcium overload. PLCγ1, IP3R, CAMKKII, and CaM proteins play important roles in mediating intracellular calcium overload and excessive accumulation of reactive oxygen species (ROS). Tongqiao Huashuan (TQHS) granules represent a formulated preparation derived from traditional Chinese medicine (TCM), which have demonstrated extensive clinical application in managing VaD. Although TQHS granules have shown favorable clinical efficacy, the underlying mechanisms remain unclear. The present investigation sought to clarify protective roles exerted by TQHS granules against VaD via modulation of relevant signaling cascades.

**Methods:**

Using a permanent bilateral common carotid artery occlusion (2-VO) rat model and an OGD/R-induced SH-SY5Y cell model, we evaluated cognitive function, neuronal injury, and pathway-related molecular changes.

**Results:**

TQHS granules markedly ameliorated behavioral deficits and restored cognitive performance in VaD rats following treatment. Immunohistochemistry and Western blotting experiments demonstrated that TQHS granules regulated protein abundance of PLCγ1, p-PLCγ1, IP3R, CAMKKII, and CaM, which are proteins related to the PLCγ1/IP3R signaling pathway, *in vivo*. Data derived from CCK-8, microplate reader detection, and immunofluorescence experiments demonstrated that TQHS granules increased cell survival rate, reduced intracellular Ca^2+^ concentration, and reduced ROS levels. RT-qPCR showed that TQHS granules increased the expression of PLCγ1, IP3R and TrkB.

**Discussion:**

The neuroprotective effect of TQHS granules on experimental VaD may be mediated by regulating the PLCγ1/IP3R pathway, improving oxidative stress injury and intracellular calcium overload in VaD. TQHS granules hold considerable promise as a viable complementary intervention capable of retarding the neurodegenerative trajectory of VaD.

## 1 Introduction

Vascular dementia (VaD) is a neurological disorder dependent on vascular pathology affecting the nervous system, and its cognitive impairment is attributed to the frequent occurrence of subcortical vascular lesions that disrupt the frontostriatal circuitry (Yang et al., 2022). VaD development is attributable to multiple predisposing conditions, notably hypertension, smoking, hypercholesterolemia, diabetes mellitus, and heart disease (Dichgans and Zietemann, 2012). VaD is the second most common type of dementia after Alzheimer’s disease, accounting for 20% of dementia cases (Akhter et al., 2021; Lecordier et al., 2021), characterized by progressive erosion of memory, thought processing, language skills, and executive functions (Mograbi et al., 2021). Recent studies indicate that dementia ranks fifth in the global disease burden, imposing an annual global economic burden of approximately US$ 1 trillion (Livingston et al., 2020), a figure projected to approach US$ 4 trillion by 2050 (Rundek et al., 2022). Among current dementia types, VaD is unique in exhibiting the feature of treatable reversibility (Wolters and Ikram, 2019). Clinically, mainstream VaD pharmacotherapy involves augmentation of cholinergic transmission and blockade of pathological glutamate signaling. Despite modest gains in intellectual capacities afforded by symptomatic treatments, VaD management remains suboptimal (Sinha et al., 2020). Therefore, identifying its therapeutic targets and exploring effective candidate drugs for VaD treatment are of significant importance.

VaD presents with multifaceted cognitive deterioration encompassing mnemonic deficits, executive function, sustained focus, verbal communication, and spatial navigation capacities (O’Brien and Thomas, 2015). Under the VasCog-2-WSO diagnostic criteria, the diagnosis of VaD requires fulfillment of the “dementia” severity criterion together with evidence that the cognitive disorder is predominantly vascular in origin, and the “probable” category further requires neuroimaging or genetic evidence of cerebrovascular disease supporting the above clinical diagnosis (VasCog-2-Wso Criteria Consortium et al., 2025). Notably, definitive identification of VaD necessitates brain imaging via documented cerebrovascular lesion load, with ischemic insults, leukoaraiosis, plus resulting structural decline offering substantiation for diagnostic formulation (Yang et al., 2023). Traditional Chinese medicine (TCM) attributes VaD to toxin-mediated collateral damage secondary to blood stasis and phlegm turbidity, categorizing it under “foolishness” and “dementia.” The collateral-unblocking therapeutic paradigm has thus emerged as a focus of clinical investigation (Zhang et al., 2023).

Tongqiao Huashuan (TQHS) granules are a modern Miao medicinal preparation for the treatment of stroke developed from TQHS decoction. Within the TQHS granules formulation, *Sargentodoxa cuneata* (Oliv.) Rehd. et Wils., *Mezoneuron cucullatum* (Roxb.) Wight & Arn., *Whitmania pigra* Whitman, and *Polygonum suffultum* Maxim. promote blood circulation and remove blood stasis; *Gastrodia elata* Bl. calms the liver and extinguishes wind to relieve spasms; *Acorus calamus* L. resolves phlegm and opens the orifices; and *Lycium barbarum* L. and *Astragalus membranaceus* (Fisch.) Bunge reinforce vital qi and tonify deficiency, thereby exerting effects of dispelling wind and resolving phlegm, activating blood and dispelling stasis, regulating qi and blood, and nourishing the liver and kidneys. Existing studies have demonstrated that gastrodin, a major active constituent of *Gastrodia elata*, alleviates oxidative injury–induced mitochondrial dysfunction and exerts neuroprotective effects in VaD (Wu et al., 2023); *Whitmania pigra* markedly inhibits platelet aggregation and prolongs coagulation time (Dong et al., 2023); and astragaloside IV from *Astragalus membranaceus* restores neurological function in VaD by restoring redox homeostasis and augmenting mature oligodendrocyte density (Ma et al., 2023). Preliminary experiments confirmed that TQHS mediated the remediation of cerebral ischemic injury manifestations in experimental stroke models via dual targeting of oxidative damage and apoptotic cascades (Cai et al., 2019), and emerging evidence indicates TQHS granules effectively enhance cognitive performance in VaD models via PI3K/Akt-mTOR axis modulation (Jiang et al., 2025).

Among the many proteins closely related to VaD, phospholipase Cγ1 (PLCγ1) and Inositol 1,4,5-trisphosphate receptor (IP3R) are particularly important, as they modulate neuronal apoptosis while affording protection to cerebral tissue (Singh et al., 2024; Luong et al., 2025), and they trigger Ca^2+^ mobilization from endoplasmic reticulum stores, thereby mitigating intracellular calcium overload and ameliorating dementia (Jang et al., 2018). Mechanistic investigations have revealed allicin to confer cardioprotection against ischemic insult via PI3K/GRK2/PLC-γ/IP3R axis regulation (Gao et al., 2021). Singh et al. described a pivotal role of PLCγ1 in neuroprotection, highlighting that it regulates neuronal survival and synaptic plasticity via Ca^2+^ signaling and constitutes a pivotal point of intervention within neurodegenerative disorders (Singh et al., 2024). However, the potential mechanism by which TQHS granules regulate the PLCγ1/IP3R pathway to treat VaD remains largely unknown. Based on this, the present investigation aimed to determine the capacity of TQHS granules to mitigate oxidative damage and alleviate intracellular calcium overload, while concurrently elucidating their potential mechanisms for improving VaD-induced cognitive impairment.

## 2 Materials and methods

### 2.1 Preparation of experimental drug

TQHS granules (20240601, Department of Pharmacy, The First Affiliated Hospital of Guizhou University of Traditional Chinese Medicine, China) and Donepezil Hydrochloride (Sibohai, National Medicine Standard H20010723, Chongqing Zhisi Pharmaceutical Co., Ltd., China) were both provided by the First Affiliated Hospital of Guizhou University of Traditional Chinese Medicine. According to the principles of “Pharmacological Experimental Methodology,” the clinical dosages of the two drugs were adjusted to dosages suitable for administration in rats. The drugs were accurately weighed and dissolved in normal saline to reach the required concentrations. These solutions were freshly prepared for oral gavage administration to the animals, and the remaining drugs were stored at 4°C for later use.

To prepare TQHS granules drug-containing serum, 20 SD rats (200 ± 20 g) were randomized into two experimental arms: TQHS granules drug-containing serum group (*n* = 10) and control serum group (*n* = 10). Following clinical dosing protocols and dose conversion algorithms based on body surface area, we derived a scaled dosage of 2.7 g/kg from human data. Animals received the TQHS granules suspension twice daily over 1 week, while controls received matching saline volumes. Whole blood withdrawn from the abdominal aorta 1 h after final dosing was clotted for 2 h at ambient temperature, then centrifuged for 15 min at 3,000 r/min. Serum was subsequently heat-inactivated at 56°C for 30 min, 0.22 μm filtered, and aliquoted for storage at −80°C.

### 2.2 Animal experiments

Adult male SD rats (aged 6–8 weeks, 200 ± 20 g) were obtained from Guizhou University of Traditional Chinese Medicine Animal Laboratory (License No.: SCXK (Qian) 2021–0003). Rats were kept in a thermally controlled housing (22 ± 1°C, 50 ± 5% humidity) under 12 h artificial lighting cycles, provided with unrestricted food and water. 48 rats were randomized into six experimental groups (*n* = 8): sham-operated control, VaD model, low-dose TQHS (1.35 g/kg), medium-dose TQHS (2.7 g/kg), high-dose TQHS (5.4 g/kg), and donepezil positive control. VaD was induced in rats via permanent bilateral common carotid artery occlusion (2-VO), following Kim et al. (2023). Animals received isoflurane (5% induction; 2% maintenance) in 21% oxygen at 2 L/min. Following cervical disinfection, the neck was opened via median incision to reveal bilateral carotid vessels, subsequently undergoing double ligation with 5–0 silk. The sham group procedures replicated all surgical steps save for bilateral carotid tying. During anesthesia, the periocular area was covered with sterile normal saline–moistened gauze to keep the corneas moist. Body temperature was maintained at 37 ± 1°C throughout the procedure. Isoflurane anesthesia was employed for all surgical manipulations, with pain management protocols implemented. The present study adhered to the ARRIVE guidelines (Animal Research: Reporting of *In Vivo* Experiments), ensuring scientific transparency. Rats in the TQHS groups and the Donepezil Hydrochloride group were administered TQHS-L (dissolved in normal saline, 1.35 g/kg), TQHS-M (dissolved in normal saline, 2.7 g/kg), TQHS-H (dissolved in normal saline, 5.4 g/kg), and Donepezil Hydrochloride (dissolved in normal saline, 0.45 mg/kg), respectively, by gavage once daily for 28 days starting 7 days after surgery. Sham-operated and model animals received matching saline volumes via daily gastric intubation for 4 weeks. The Animal Ethics Committee of Guizhou University of Traditional Chinese Medicine approved this protocol on October 28, 2024 (Ethics Approval No. 2024083); experiments adhered to applicable ethical guidelines.

### 2.3 Morris water maze

Rats were tested 7 days after modeling and at the end of drug administration. Localization and navigation experiment: A platform was placed in any quadrant, and animals were released from varying start locations (one per quadrant) with orientation toward the pool wall, over 4 consecutive acquisition days. Escape latency was defined as the time required to locate the platform (maximum 90 s), animals failing to locate the target were manually placed on it (10 s) and assigned 90-s latency. The mean value of each quadrant on the 4th day was taken as the final result of the escape latency of the rats. Spatial Probe: Following platform removal, animals were released from the quadrant opposite the former target location and platform crossings (≤ 90 s) were tallied (Morris, 1984; Liu and Borisyuk, 2024).

### 2.4 Novel object recognition

The NOR test was performed at the end of drug administration. Throughout the 10-min acquisition session, we quantified investigation time for a pair of identical objects. After 24 h, one object was replaced by a novel counterpart matching in size yet differing in color and shape. Novel and familiar object exploration was documented over a 5-min period. We evaluated recognition memory based on differential exploration of novel versus familiar objects (Seyedhosseini Tamijani et al., 2023). Object recognition performance was scored using the discrimination index (DI). DI = (time spent with novel object − time spent with familiar object)/total exploration time, which was used to evaluate the cognitive ability and memory level of old and new things in rats.

### 2.5 Sample collection

Following behavioral assessments, subjects received anesthesia (5% isoflurane induction followed by 2% maintenance) for terminal cardiac blood collection. Following 2-h settling at 4°C and centrifugation (3,000 rpm, 5 min, 4°C), the resulting supernatant was cryopreserved at −80°C. Following blood collection, once the rats were confirmed to be in deep anesthesia with loss of the pedal withdrawal reflex and corneal reflex, they were euthanized by cervical dislocation. Brain tissue was rapidly removed on an ice plate, with a portion stored at −80°C and another portion fixed in paraformaldehyde.

### 2.6 Immunohistochemistry

Brain coronal sections were blocked in 10% goat serum (C0265, Beyotime Biotech Inc., China) at ambient temperature for 1 h, subsequently incubated overnight at 4°C with primary antibodies targeting PLCγ1 (1:200 dilution, AF6210, AB_2835091, Affinity Biosciences, United States) and CaM (1:200 dilution, AF6353, AB_2835158, Affinity Biosciences, United States). After incubation with secondary antibodies (1:500 dilution, GB23303, AB_2811189, Servicebio, China) for 40 min, immunohistochemical staining was visualized using a 3,3′-diaminobenzidine (DAB) kit (SW01278, Shenzhen Sunview Technology Co., Ltd., China) to visualize antigen–antibody complexes. Sections were counterstained with Hematoxylin stain (SW01892, Shenzhen Sunview Technology Co., Ltd., China). Digital image analysis (ImageJ) quantified positive-staining area and IOD, yielding relative protein expression (mean optical density = IOD/area) (Tian et al., 2021).

### 2.7 Western blot

Hippocampal tissue retrieved from −80°C was homogenized, and the lysate underwent protein extraction. Protein concentration was quantified via BCA assay (B6169, UElandy, China), and 50 μg per sample was resolved by SDS-PAGE, transferred to membranes, and blocked with 5% skim milk following TBST washes. Following washes and blocking (5% skim milk, 1 h), membranes were probed overnight (4°C) with primary antibodies against p-PLCγ1 (1:2,000 dilution, AF3210, AB_2834502, Affinity Biosciences, United States), PLCγ1 (1:2,000 dilution, AF6210, AB_2835091, Affinity Biosciences, United States), IP3R (1:2000 dilution, DF3000, AB_2840979, Affinity Biosciences, United States), CAMKKII (1:1,000 dilution, DF4793, AB_2837147, Affinity Biosciences, United States), GAPDH (1:50,000 dilution, 60004-1-Ig, AB_2107436, Proteintech, China), CaM (1:2,000 dilution, AF6353, AB_2835158, Affinity Biosciences, United States) and β-actin (1:10,000 dilution, AF7018, AB_2839420, Affinity Biosciences, United States). Secondary antibody incubation (ambient temperature, 60 min) was followed by chemiluminescent visualization using ECL reagent (BL520B, Biosharp, China). ImageJ densitometry was used to quantify band intensities, with values normalized to β-actin or GAPDH for relative protein expression. Each sample was analyzed separately in triplicate.

### 2.8 OGD/R model and drug treatments

OGD/R injury was induced in SH-SY5Y cells (Homo sapiens, neuroblastoma of the brain with metastasis to bone marrow, CVCL-0019, CL-0208) by 4 h exposure to hypoxia (5% CO2/95% N2) in glucose- and serum-free medium. Thereafter, cultures were transferred to complete DMEM supplemented with 10% FBS and maintained for 24 h in normoxia.

To evaluate U73122 (HY-13419, Med Chem Express, China) effects on normal SH-SY5Y cells, we established: control and U73122 groups at concentrations of 0.1, 0.5, 1, 5, 10, 20, 40, 80, and 100 μmol/L. Subsequently, to elucidate U73122-mediated protection against OGD/R injury via the PLCγ1/IP3R axis in SH-SY5Y cells, we established: control group, OGD/R model, 5, 10, 20, and 40 μmol/L U73122 groups. All groups except controls underwent OGD/R insult, subsequently receiving U73122 intervention across a dose range prior to reperfusion.

To elucidate mechanisms of TQHS-containing serum against OGD/R injury via the PLCγ1/IP3R pathway in SH-SY5Y cells, groups included: control, OGD/R model, 10% control serum, TQHS at 10, 15, and 20%, and U73122-treated groups. All cohorts except control were subjected to OGD/R; the 10% control group received equivalent serum volumes, while treatment groups were reperfused with respective concentrations.

### 2.9 Cell viability assay

We evaluated cell survival via CCK-8 assay (CCK-8, C0038, Beyotime Biotech Inc., China). SH-SY5Y cells received 10 μL CCK-8 reagent and were maintained (37°C, dark, 1–2 h) before spectrophotometric detection at 450 nm. Viability was normalized to the control group (Duan et al., 2019).

### 2.10 The levels of calcium ion (Ca^2+^) and reactive oxygen species release

SH-SY5Y cells were harvested, washed three times with PBS for 5 min each, and loaded with Fluo-4 AM per the instructions of the Fluo-4 Calcium Ion Assay Kit (S1061S, Beyotime Biotech Inc., China). Following 30 min incubation and one to three additional PBS washes, intracellular Ca^2+^ fluorescence was quantified using a fluorescence microplate reader.

Following identical washing, ROS levels in SH-SY5Y cells were assessed using the fluorescent probe 2′,7′-dichlorodihydrofluorescein diacetate (DCFH-DA) per the instructions of the ROS Assay Kit (S0034S, Beyotime Biotech Inc., China). Fluorescence images were captured using microscopy, and signal intensity was quantified via Image J.

### 2.11 RT-qPCR analysis

Total RNA was extracted from SH-SY5Y cells via the Trizol method according to the manufacturer’s instructions. cDNA was reverse-transcribed using the RevertAid First Strand Kit (K1622, Thermo Fisher Scientific, United States) and subjected to quantitative PCR (40 cycles: 95°C/10 min; 95°C/15 s; 60°C/1 min) on a real-time fluorescence detection system. Expression levels were normalized to β-actin (endogenous control) using the 2^–△△^*^CT^* method. All reactions were run in technical triplicate. Primers (Shanghai Generay Biotech Co., Ltd., China) for TrkB, PLCγ1, IP3R, and β-actin appear in Supplementary Table 1.

### 2.12 Statistical analysis

Data analysis was performed using SPSS 26.0. Results were presented as mean ± SD. MWM data were subjected to two-way repeated-measures ANOVA, whereas other outcomes were evaluated via one-way ANOVA with Tukey’s *post hoc* multiple comparisons. For data exhibiting heteroscedasticity, non-parametric alternatives were applied. Significance was set at *P* < 0.05.

## 3 Results

### 3.1 TQHS granules rescued VaD rats from cognitive decline

**FIGURE 1:**
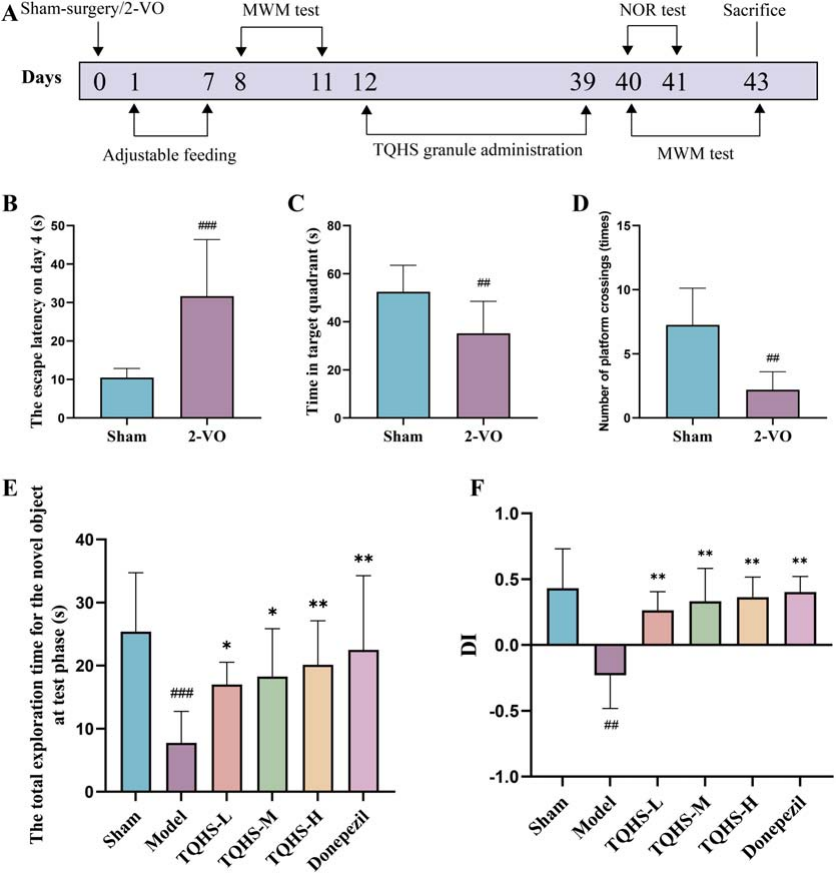
TQHS granules ameliorated cognitive deficits and enhanced memory abilities in VaD rats. **(A)** Schematic timeline of the animal study. **(B–D)** The MWM test after day 7 of VaD modeling. **(B)** The escape latency for reaching the hidden platform on day 4 in the probe navigation trial. **(C)** The time spent in the target quadrant in the spatial probe trial. **(D)** The number of platforms crossings in the spatial probe trial. **(E)** The total exploration time for the novel object at test phase. **(F)** Discrimination index (DI) of NOR test. Values were expressed as means ± SD (*n* = 8). ^##^*P* < 0.01, ^###^*P* < 0.001 versus sham-surgery group. *P < 0.05, **P < 0.01 versus model group.

To determine whether TQHS granules could ameliorate VaD, the NOR and MWM tests were performed after 28 days of administration (Figure 1A) to assess cognitive memory performance. Serving as a conventional tool for the investigation of spatial cognition in rats, the Morris water maze captures escape latency, crossing frequency, and target quadrant dwelling time as core metrics (Tucker et al., 2018). Following 2-VO surgery, the VaD-model rats exhibited markedly extended escape latency on day 4 (^###^*P* < 0.001, vs. Sham group) (Figure 1B), reduced time spent in the target zone (^##^*P* < 0.01, vs. Sham group) (Figure 1C), and decreased platform crossings (^##^*P* < 0.01, vs. Sham group) (Figure 1D), indicating impaired cognitive performance in VaD rats. On day 2 of the NOR test, the model animals showed markedly reduced total exploration time of the novel object relative to the sham group. In contrast, both the TQHS-treated and donepezil-treated VaD rats exhibited longer total exploration times in the NOR test relative to the model animals, and the effect of TQHS granules was dose-dependent (*P < 0.05, **P < 0.01, vs. Model group) (Figure 1E). VaD rats demonstrated substantially lower DI levels relative to sham-operated animals; however, this impairment was markedly reversed following intervention with TQHS or donepezil (**P < 0.01, vs. Model group) (Figure 1F). Current findings demonstrate that both TQHS granules and donepezil significantly restore recent memory function among VaD rats.

**FIGURE 2:**
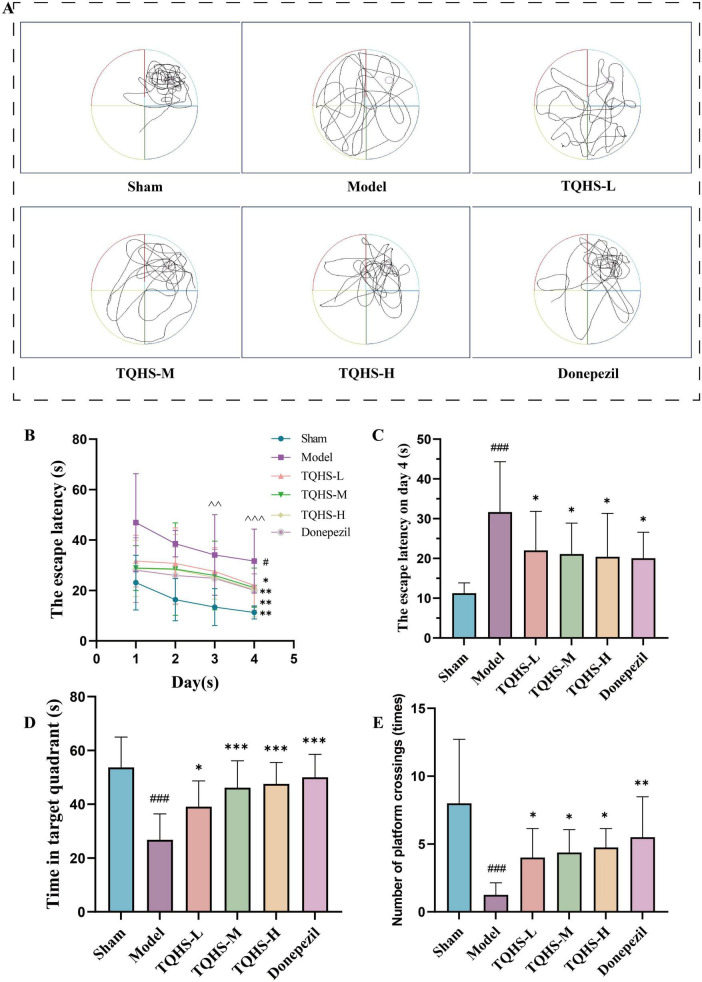
TQHS granules ameliorated cognitive deficits and enhanced memory abilities in VaD rats. **(A–E)** The MWM test 28 days after the administration of TQHS granules. **(A)** Representative swimming trace in the spatial probe trial. **(B)** The escape latency for reaching the hidden platform during 4 days in the probe navigation trial. **(C)** The escape latency for reaching the hidden platform on day 4 in the probe navigation trial. **(D)** The time spent in the target quadrant in the spatial probe trial. **(E)**-the number of platforms crossings in the spatial probe trial. Values were expressed as means ± SD (*n* = 8). ^#^*P* < 0.05, ^###^*P* < 0.001 versus sham-surgery group. *P < 0.05, **P < 0.01, ***P < 0.001, versus model group. ^∧∧^ *P* < 0.01, ^∧∧∧^ *P* < 0.001 versus the escape latency on day 1.

After 28 days of treatment, escape latency in the VaD-model group was markedly longer than in the sham group on days 1, 2, 3, and 4 of the spatial navigation training, while rats in the TQHS and donepezil groups exhibited markedly shorter escape latencies than those in the VaD-model group (*P < 0.05, **P < 0.01, vs. Model group) (Figure 2B). Moreover, relative to day 1, the escape latency on days 3 and 4 was markedly reduced in all groups, indicating progressive learning over time. On day 4, the escape latency remained markedly longer in the VaD-model group relative to the sham group, while TQHS granules or donepezil administration markedly attenuated this prolongation (*P < 0.05, vs. Model group) (Figure 2C). After the hidden platform was removed, rats in the model group spent significantly less time in the target zone relative to the sham group, whereas TQHS granules and donepezil treatment markedly increased the time spent in the target zone (*P < 0.05, ***P < 0.001, vs. Model group) (Figures 2A,D). The number of platform crossings was also markedly lower in the model group, but increased after treatment with TQHS granules or donepezil (*P < 0.05, **P < 0.01, vs. Model group) (Figure 2E). These findings demonstrate that TQHS granules and donepezil confer comparable benefits on cognitive performance in VaD models, with TQHS effects exhibiting dose-dependency.

### 3.2 TQHS granules activate the PLCγ1/IP3R pathway in the hippocampus of VaD rats

**FIGURE 3:**
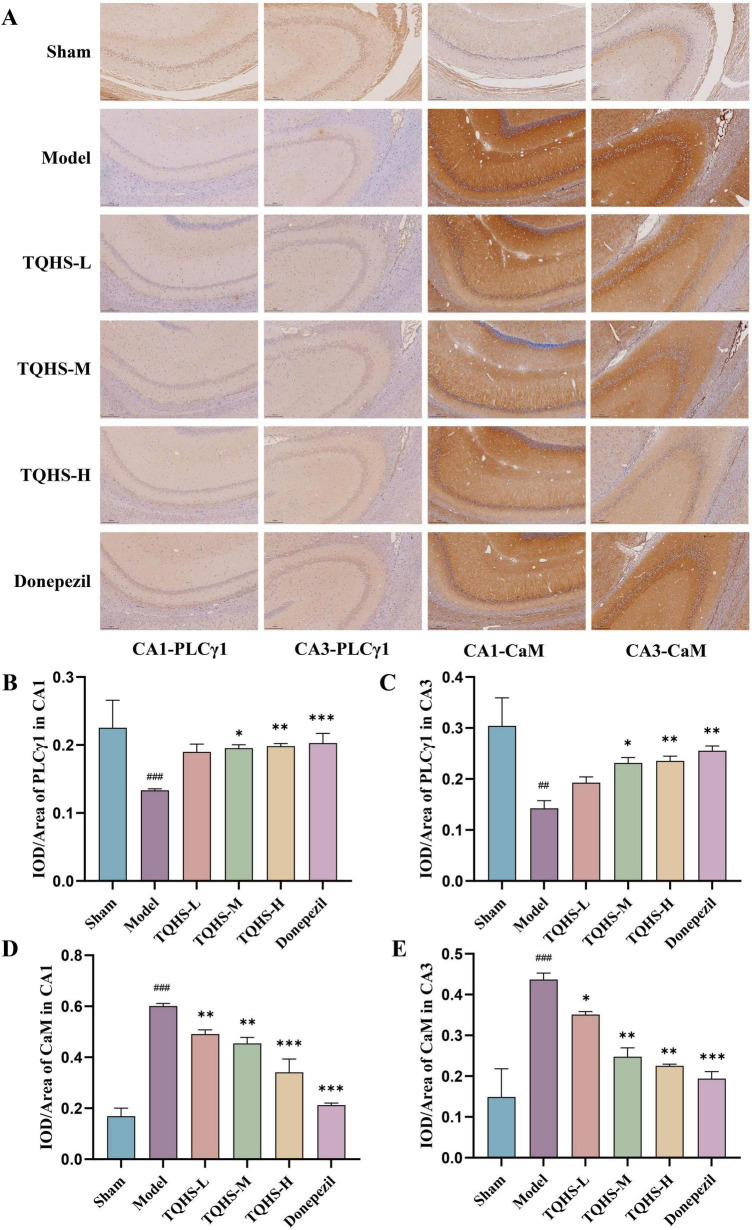
TQHS increased PLCγ1 expression, whereas reduced CaM expression in the hippocampal CA1 and CA3 regions of VaD Rats. **(A)** Immunohistochemical analysis of PLCγ1 and CaM expression in hippocampal CA1 and CA3 regions (× 10) (*n* = 2). **(B–E)** Quantitative analysis showed the expression of **(B)** PLCγ1, and **(D)** CaM in hippocampal CA1 regions and **(C)** PLCγ1, and **(E)** CaM in hippocampal CA3 regions. Values were expressed as means ± SD (*n* = 3). ^##^*P* < 0.01, ^###^*P* < 0.001 versus sham-surgery group. *P < 0.05, **P < 0.01, ***P < 0.001 versus model group.

Immunohistochemical analysis of the hippocampus revealed that PLCγ1 levels in the CA1 and CA3 regions were remarkably declined in the VaD-model group, whereas its reduction was markedly attenuated in the TQHS granules and donepezil treatment groups (*P < 0.05, **P < 0.01, ***P < 0.001, vs. Model group) (Figures 3A–C). Hippocampal cells of the sham group exhibited normal staining without brown granule deposition. Relative to the sham group, the VaD-model group exhibited markedly attenuated nuclear staining, increased brown or yellowish-brown granules, and a higher positive expression rate of CaM in the CA1 and CA3 regions. TQHS granules administration and donepezil both reduced granule accumulation and the positive expression of CaM in a dosage-proportional fashion (*P < 0.05, **P < 0.01, ***P < 0.001, vs. Model group) (Figures 3A–E).

**FIGURE 4:**
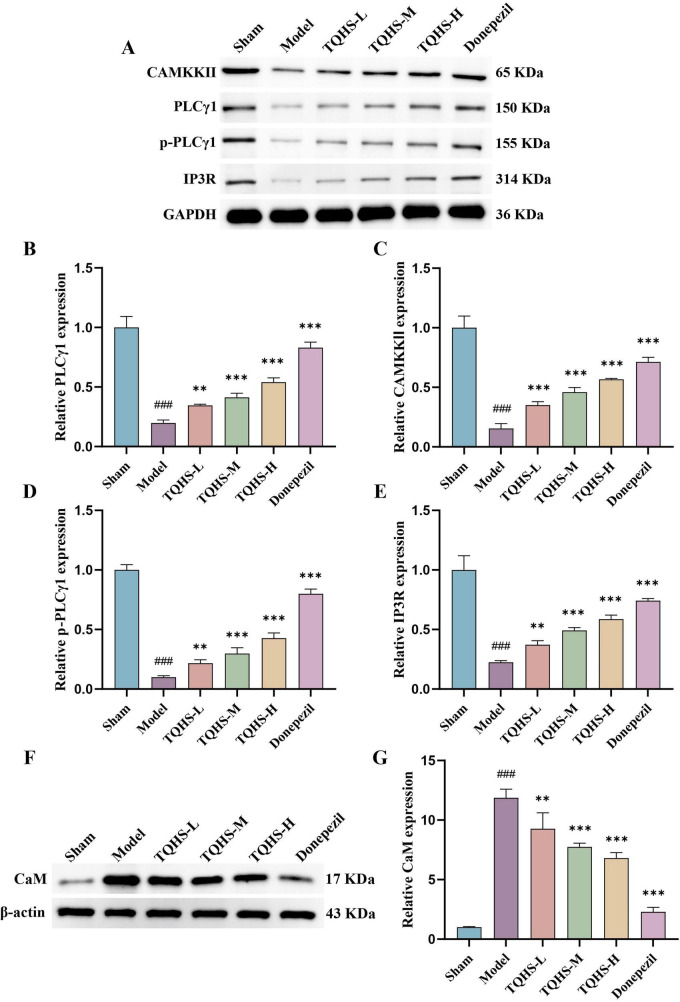
TQHS increased PLCγ1, p-PLCγ1, IP3R and CAMKKII expression, whereas reduced CaM expression in the hippocampus of VaD rats. **(A,F)** Western blot was performed to detect the levels of PLCγ1, p-PLCγ1, IP3R, CAMKKII and CaM in the hippocampus of VaD rats. Quantitative analysis of **(B)** PLCγ1, **(C)** CAMKKII, **(D)** p-PLCγ1, **(E)** IP3R and **(G)** CaM expression. The densities of the bands were normalized with respect to the values of GADPH and β-actin. Values were expressed as means ± SD (*n* = 3). ^###^*P* < 0.001 versus sham-surgery group. **P < 0.01, ***P < 0.001 versus model group.

WB analysis of PLCγ1/IP3R pathway-related proteins revealed that PLCγ1, phosphorylated PLCγ1 (p-PLCγ1), IP3R, and CAMKKII expression was remarkably decreased in the VaD-model group, whereas treatment with TQHS granules or donepezil increased their expression (**P < 0.01, ***P < 0.001 vs. Model group) (Figures 4A–E). Moreover, CaM expression was markedly elevated in the VaD-model group relative to the sham group but notably reduced following treatment with TQHS granules or donepezil (**P < 0.01, ***P < 0.001 vs. Model group) (Figures 4F,G). Collectively, these results support that TQHS granules improve VaD via PLCγ1/IP3R pathway activation.

### 3.3 TQHS granule-containing serum protects SH-SY5Y cells from OGD/R-induced injury by downregulating ROS and Ca^2+^ levels

**FIGURE 5:**
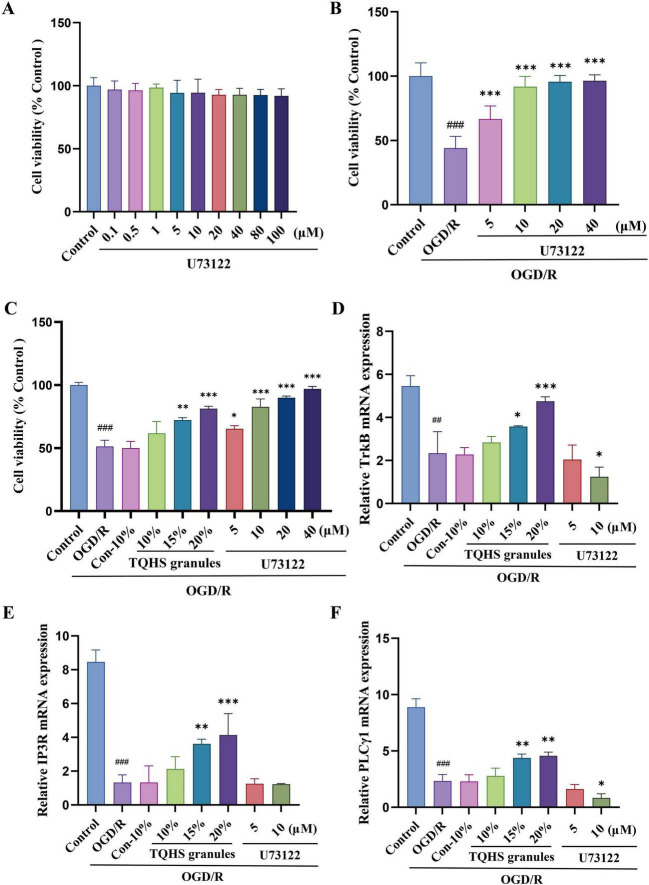
Effects of TQHS granules on OGD/R-induced cytotoxicity SH-SY5Y cells and the mRNA expressions of PLCγ1, IP3R, and TrkB. **(A)** Effects of U73122 on normal SH-SY5Y cell viability. **(B,C)** Effects of B-U73122 and **(C)** TQHS granules on OGD/G cell viability. **(D–F)** RT-qPCR analyzed **(D)** TrkB, **(E)** IP3R and **(F)** PLCγ1 mRNA expression levels. Values were expressed as means ± SD (*n* = 3). ^##^*P* < 0.01, ^###^*P* < 0.001 versus control group. *P < 0.05, **P < 0.01, ***P < 0.001 versus OGD/R group.

To explore the effects of TQHS granule-containing serum on OGD/R-induced SH-SY5Y cell injury, the PLC inhibitor U73122 was used as a positive control. CCK-8 assays showed that treatment with increasing concentrations of U73122 failed to alter the proliferative capacity of normal SH-SY5Y cells (Figure 5A). Conversely, when SH-SY5Y cells were subjected to OGD/R followed by U73122 treatment, cell viability significantly improved at concentrations of 5, 10, 20, and 40 μmol/L (***P < 0.001, vs. OGD/R group) (Figure 5B). Furthermore, treatment with TQHS-containing serum significantly increased the survival rate of OGD/R-induced SH-SY5Y cells relative to the OGD/R group (**P < 0.01, ***P < 0.001, vs. OGD/R group) (Figure 5C).

**FIGURE 6:**
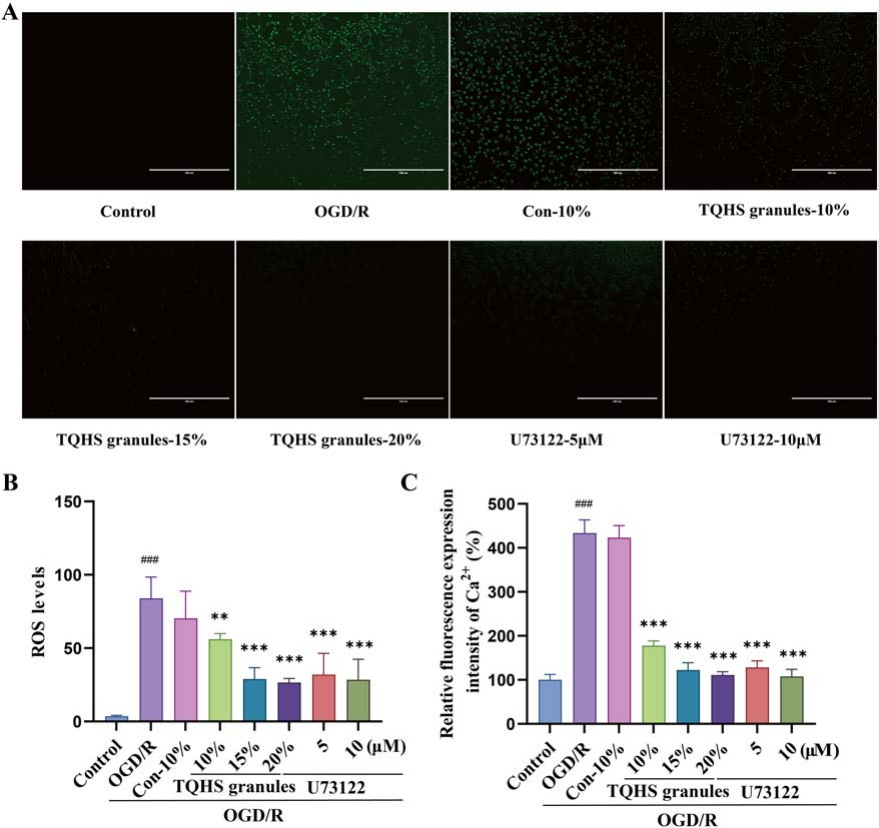
Effects of TQHS granules on the levels of ROS in SH-SY5Y cells by using immunofluorescence and the levels of Ca^2+^ in SH-SY5Y cells by using the microplate reader. **(A)** Representative photomicrographs of ROS immunofluorescence are shown. **(B,C)** The graphs show the levels of ROS and Ca^2+^ in SH-SY5Y cells, the mean fluorescence intensity represents the level of ROS and Ca^2+^. Values were expressed as means ± SD (*n* = 3). ^###^*P* < 0.001 versus control group. **P < 0.01, ***P < 0.001 versus OGD/R group.

To assess the antioxidant capacity exerted by TQHS granule-containing serum, oxidative stress markers were assessed. The OGD/R group exhibited significantly elevated ROS accumulation, which was markedly reduced following treatment with TQHS-containing serum or U73122 in a dosage-proportional fashion (**P < 0.01, ***P < 0.001, vs. OGD/R group) (Figures 6A,B). Similarly, intracellular Ca^2+^ levels were strikingly elevated in the OGD/R group relative to the control group, whereas treatment with TQHS-containing serum or U73122 significantly decreased Ca^2+^ levels in a dosage-proportional fashion (***P < 0.001, vs. OGD/R group) (Figure 6C).

### 3.4 TQHS granule-containing serum alleviates OGD/R-induced injury in SH-SY5Y cells via the PLCγ1/IP3R signaling pathway

To further examine the regulatory effect of TQHS on the PLCγ1/IP3R pathway *in vitro*, the mRNA levels of TrkB, PLCγ1, and IP3R were measured in OGD/R-stimulated SH-SY5Y cells. RT-qPCR analyses revealed that all three mRNA expression levels were downregulated in the OGD/R group relative to the control group. However, treatment with 15 and 20% TQHS granule-containing serum prominently upregulated the mRNA expression of these three genes, while 10 μmol/L U73122 significantly lowered the mRNA levels of PLCγ1 and TrkB (*P < 0.05, **P < 0.01, ***P < 0.001, vs. OGD/R group) (Figures 5D–F).

## 4 Discussion

VaD constitutes a serious cognitive impairment syndrome induced by ischemic stroke, hemorrhagic stroke, or chronic cerebral hypoperfusion (CCH), thereby triggering oxidative stress and neurotoxic responses (Wang et al., 2021). In clinical practice, VaD interventions focus on boosting cerebral blood flow and perfusion, reducing cerebral infarction, and enhancing brain metabolism to relieve symptoms and slow disease progression. Cerebral hypoperfusion, oxidative stress injury, and intracellular calcium overload are considered key pathogenic mechanisms associated with VaD (Van Cauwenberghe et al., 2016). Earlier investigations indicate the PLCγ1/IP3R pathway can reduce endoplasmic reticulum calcium release after reperfusion, attenuate mitochondrial ROS production, along with prevent mitochondrial membrane potential (MMP) loss. However, the relationship among the PLCγ1/IP3R signaling pathway, neuronal apoptosis, inflammatory responses, and VaD remains largely unclear. TQHS granules inhibit hippocampal neuronal autophagy in VaD through PI3K/Akt-mTOR signaling pathway activation. However, how their effects correlate with oxidative stress and intracellular calcium overload is yet to be elucidated. Therefore, we hypothesize that the cognitive-enhancing and neuroprotective effects of TQHS granules may be attributed to their antioxidative properties and their ability to reduce intracellular calcium overload. To clarify these issues, we measured oxidative stress and intracellular calcium levels and investigated the underlying mechanisms across *in vivo* and *in vitro* systems.

The hippocampus is essential for information processing and storage, governing sophisticated neural operations, including learning and memory (Treder et al., 2021). Accumulated evidence indicates that ischemic brain damage stemming from CCH constitutes an important pathological basis for the development and progression of VaD. As pathological changes unfold, inadequate oxygen and glucose supply leads to a series of homeostatic disturbances, including oxidative stress, apoptosis, autophagy, and intracellular calcium imbalance. CCH induces apoptosis, enhanced and redistributed autophagy of hippocampal neurons in rats (Zou et al., 2018), and the number of surviving hippocampal neurons directly affects hippocampal information processing and storage (Ofori et al., 2019). Research findings indicate that TCM exerts a significant effect in alleviating hippocampal neuronal injury in VaD rats and improve their spatial cognitive abilities (Bai and Zhang, 2021). Therefore, this study employed the classic VaD animal model established by 2-VO surgery to investigate hippocampal neuronal injury, which is closely associated with cognitive functions (Jearjaroen et al., 2024; Ningning et al., 2024).

**FIGURE 7:**
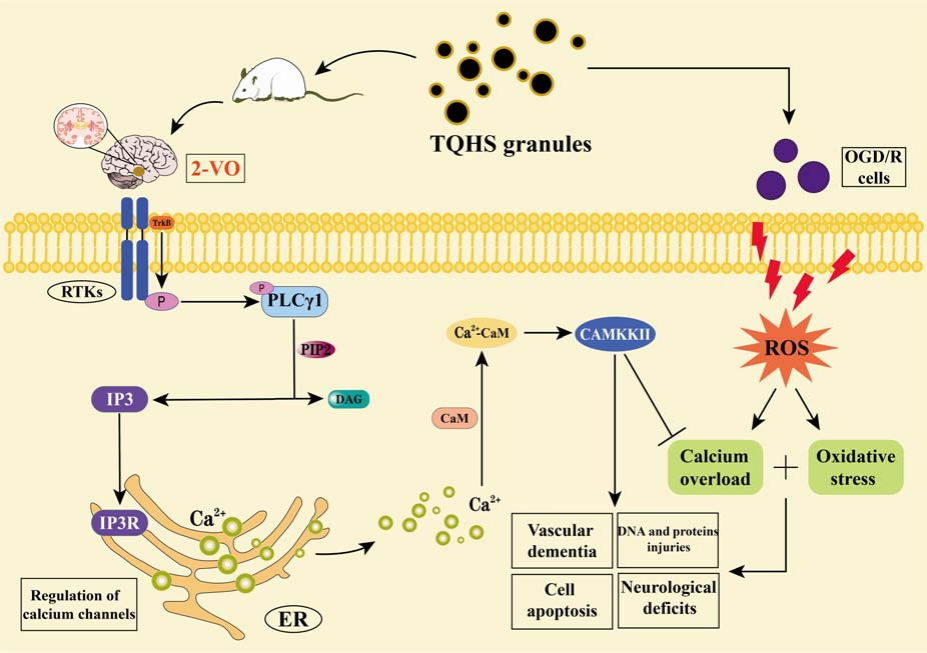
Potential mechanism underlying the neuroprotective effects of TQHS granules in VaD rats and SH-SY5Y cells. TQHS granules improved cognitive function in VaD rats, generated IP3 through PLCγ1-mediated catalytic hydrolysis of PIP2, and then affected the expression of IP3R downstream of the pathway, thus protecting the brain from VaD-induced oxidative stress and calcium overload, and further induced antioxidant activity and regulated intracellular calcium overload mechanisms.

Earlier investigations have documented therapeutic benefits conferred by TQHS granules on VaD rats. PLCγ1, an important mediator of transmembrane signaling, is highly expressed in brain tissue (Yang et al., 2016) and functions as an essential mediator of cellular signal transduction (Jang et al., 2018). PLCγ1-mediated cleavage of phosphatidylinositol 4,5-bisphosphate (PIP2) gives rise to diacylglycerol plus inositol 1,4,5-trisphosphate (IP3) (Fukami et al., 2010), which binds to its intracellular receptor IP3R, induces ligand-gated Ca^2+^ channel opening in the endoplasmic reticulum, increases intracellular Ca^2+^ levels (Wang et al., 2019), and regulates multiple pathways such as Ca^2+^/CaM/CAMKKII (Figure 7; Yang et al., 2017). Among the EF-hand class of Ca^2+^-binding proteins, CaM stands as the most thoroughly investigated and archetypal example. When intracellular Ca^2+^ concentrations increase, CaM exhibits enhanced binding affinity. Excess intracellular Ca^2+^ binds to CaM to form the Ca^2+^-CaM complex, which subsequently activates downstream substrate CAMKKII and directly or indirectly participates in protein phosphorylation (Chin and Means, 2000), thereby contributing to the progression of diverse pathological conditions (Swarnkar et al., 2012). In rodent models of Huntington’s disease, hippocampal levels of PLCγ1 and its phosphorylated form exhibit significant downregulation (Giralt et al., 2009). Our experiments confirmed similar findings within VaD rat hippocampi, demonstrating TQHS granules treatment restored the expression of PLCγ1 and p-PLCγ1. As a downstream effector of PLCγ1, IP3R orchestrates the liberation of Ca^2+^ from endoplasmic stores and is abundantly localized to neuronal cells (Sharp et al., 1999). Prior research has revealed that the PLC/IP3R/CAMKKII axis contributes to the activation of downstream AMPK/GSK-3β/Nrf2 signaling, thereby exerting neuroprotective effects in ischemic stroke, in which CAMKKII levels are reduced. These findings suggest that CAMKKII can mediate multiple signaling pathways to ameliorate neurodegeneration (Yuan et al., 2022; Kaur et al., 2024; Zhang et al., 2025). Acting as a serine/threonine kinase, CAMKKII conveys downstream signals from hormones, metabolites, inflammatory factors, as well as neuroendocrine inputs, regulating cognition and neural development (Najar et al., 2021; Wani et al., 2021), while attenuating neuronal apoptosis and injury (Lee et al., 2022). According to Qu et al. (2021), mitochondrial biogenesis as well as glucose uptake in myotubes is mediated by transcription factors via the Ca^2+^ influx-induced CAMKKII/AMPK signaling cascade. In this study, concordant alterations in hippocampal CaM and Ca^2+^ levels were observed among VaD rats. TQHS granules treatment attenuated the reduction of CAMKKII expression and the elevation of CaM expression. Elevated hippocampal expression of PLCγ1, p-PLCγ1, IP3R, plus CAMKKII among VaD rats suggests neuroprotection by TQHS granules potentially involves PLCγ1/IP3R signaling pathway.

Redox homeostasis disruption characterizes cerebral ischemia and critically underlies VaD pathophysiology (Yang and Zhang, 2021). Brain ischemia induced by vascular injury alters circulating glucose levels and elevates ROS, thereby promoting redox imbalance (Warren et al., 2014). Oxidant overload promotes vascular endothelium impairment and functional deterioration (Chrissobolis et al., 2012; Gray and Jandeleit-Dahm, 2015), and their disproportion markedly accelerates dementia-related neurodegeneration (Bennett et al., 2009). Brain hypoperfusion predisposes to defective mitochondrial function and metabolic crisis, constituting nascent hallmarks of neurodegenerative conditions as seen in VaD (Esteras et al., 2016). The mitochondrial respiratory apparatus governs both cellular energy status and oxidant homeostasis. Collapse of oxidative phosphorylation and the electron transport system triggers overwhelming free radical formation. Prolonged exposure to elevated ROS undermines organelle performance, resulting in hypoxic injury and loss of cell viability (Venkat et al., 2015). Additionally, mitochondrial compromise triggers excitotoxic mechanisms via depletion of intracellular ATP reserves as well as induction of calcium overload (Matuz-Mares et al., 2022). These organelles rely upon the membrane potential gradient to rapidly absorb cytoplasmic Ca^2+^ via mitochondrial uniporters. Dysfunction of mitochondrial ETC results in ROS formation, damages mitochondrial membranes, and disrupts the Ca^2+^ sequestration and accumulation, consequently increasing intracellular Ca^2+^ accumulation and aggravating calcium-dependent neurotoxicity (Carbone et al., 2017). Studies have confirmed that intracellular calcium homeostasis imbalance is also closely associated with brain ischemia-induced VaD (Moreno-Rodríguez et al., 2025), and Ca^2+^ dysregulation serves as a pivotal mechanism underlying ischemic neurodegeneration (Rahi and Kaundal, 2024). Tian et al. (2022) found that VaD-induced spatial and recognition memory deficits in rats potentially stems from tissue Ca^2+^ accumulation, ending in neuronal mortality via calcium toxicity. In this study, we demonstrated that TQHS granule-containing medicated serum enhanced cellular survival while mitigating both apoptosis and intracellular ROS and Ca^2+^ overloads, yielding neuroprotection against OGD/R-induced injury. Such observations indicate neuroprotection conferred by TQHS granules may be mediated by their antioxidant properties and regulation of intracellular calcium levels.

Use-dependent discharge of various ligands outside the cell, including neuromediators, growth-promoting proteins, and systemic hormones, influences neural ontogeny, synaptic efficacy, and cerebral pathologies (Flavell and Greenberg, 2008). They engage the PLCβ (1–4) and PLCγ (1–2) subfamilies via G protein-coupled receptors (GPCRs) and receptor tyrosine kinases (RTKs) signaling (Yang et al., 2013; Follo et al., 2015). TrkB is a member of RTK superfamily and a high-affinity receptor for brain-derived neurotrophic factor (Enkavi et al., 2024). Upon activation, this receptor activates the PLCγ-Ca^2+^ pathway through downstream signaling (Arévalo and Deogracias, 2023; Li et al., 2024). Activated PLCγ hydrolyzes PIP2 into the secondary mediators diacylglycerol and IP3, crucial for intracellular signaling (Fukami et al., 2010). Our study found that activation of the PLCγ1/IP3R signaling pathway reduces intracellular Ca^2+^ levels and functions pivotally in safeguarding neurons against injury, providing an actionable target against brain damage.

This investigation is not without limitations. First, future work will employ liquid chromatography–mass spectrometry (LC–MS) to further characterize the bioactive components within TQHS granules that enter the circulation and to elucidate their pharmacologically active material basis. Second, the conclusion that TQHS granules modulate the PLCγ1/IP3R pathway still lacks further *in vivo* validation using pathway-specific inhibitors and agonists to strengthen the current findings. Finally, to substantiate the systems pharmacology paradigm and elucidate the pleiotropic mechanisms underlying traditional herbal medicines, subsequent studies will use the bioactive constituents of TQHS granules to interrogate key targets in greater depth.

## 5 Conclusion

In summary, we demonstrated that TQHS granules can improve cognitive function in VaD and protect the rat brains as well as SH-SY5Y cells of VaD from OGD/R insult *in vivo* and *in vitro*. Current data suggest that these effects are mediated by IP3R receptor binding activation, a downstream protein of PLCγ1, which further induces antioxidant activity and modulates intracellular calcium overload mechanisms. Therefore, TQHS granules are capable of ameliorating VaD-associated oxidative stress and calcium overload pharmacologically. These findings enhance our understanding of the mechanistic involvement of TQHS granules and the PLCγ1/IP3R pathway in VaD.

## Data Availability

The original contributions presented in the study are included in the article/Supplementary material, further inquiries can be directed to the corresponding authors.
