# Supplementary material for: Exploring the neuroprotective mechanism of Tongqiao Huashuan granules in vascular dementia based on PLCγ1/IP3R signaling pathway
Source: Front Aging Neurosci. 2026 Mar 4;18:1719664. doi: 10.3389/fnagi.2026.1719664 (PMC12995809; doi:10.3389/fnagi.2026.1719664)
Supplement: Supplementary file 1 [file Table_1.docx]

Supplementary Material

# 1 Supplementary Table 1 Primer sequences

| **Primer name** |  | **Sequence (5′–3′)** |
| --- | --- | --- |
| TrkB | F | CCACGGATGTTGCTGACCAAAG |
| TrkB | R | GCCAAACTTGGAATGTCTCGCC |
| PLCγ1 | F | CTGTTCCACAGACGAATGCCCA |
| PLCγ1 | R | GAGTTAGGCTCATTGCGTTTCCG |
| IP3R | F | GCAACCACATCTGGACGCTCTT |
| IP3R | R | AGAAGGCACTGATGGTGTCCAG |
| β-actin | F | GGAGATTACTGCCCTGGCTCCTAGC |
| β-actin | R | GGCCGGACTCATCGTACTCCTGCTT |

# 2 Supplementary Table 2 Information about the antibodies

| Species | Name | Sequence | Dilution | RRID | Catalogue Number | Supplier |
| --- | --- | --- | --- | --- | --- | --- |
| Rabbit | beta Actin Antibody | / | 1:10000 | AB_2839420 | AF7018 | Affinity Biosciences |
| Rabbit | Calmodulin1/2/3 Antibody | / | 1:2000 | AB_2835158 | AF6353 | Affinity Biosciences |
| Rabbit | CAMKK2 Antibody | / | 1:1000 | AB_2837147 | DF4793 | Affinity Biosciences |
| Rabbit | PLCG1 Antibody | / | 1:2000 | AB_2835091 | AF6210 | Affinity Biosciences |
| Rabbit | Phospho-PLCG1 (Tyr783) Antibody | / | 1:2000 | AB_2834502 | AF3210 | Affinity Biosciences |
| Rabbit | IP3 Receptor Antibody | / | 1:2000 | AB_2840979 | DF3000 | Affinity Biosciences |
| Mouse | GAPDH Monoclonal antibody (1E6D9) | / | 1:50000 | AB_2107436 | 60004-1-Ig | Proteintech |
| Goat | HRP-conjugated Goat Anti-Rabbit IgG(H+L) | / | 1:10000 | AB_2722564 | SA00001-2 | Proteintech |
| Goat | HRP-conjugated Goat Anti-Mouse IgG(H+L) | / | 1:10000 | AB_2722565 | SA00001-1 | Proteintech |
| Goat | HRP-conjugated Goat Anti-Rabbit IgG(H+L) | / | 1:500 | AB_2811189 | GB23303 | Servicebio |

# 3 Supplementary Table 3 Suppliers of materials and reagents with catalog numbers

| Materials and reagents | Catalogue Number | Supplier |
| --- | --- | --- |
| Donepezil Hydrochloride（Sibohai） | H20010723 | Chongqing Zhisi Pharmaceutical Co., Ltd. |
| TQHS granules | 20240601 | the Pharmacy Department of the First Affiliated Hospital of Guizhou University of Traditional Chinese Medicine |
| U-73122 | HY-13419 | Med Chem Express |
| Isoflurane (for pets) | 2024081901 | RWD Life Science Co., Ltd. |
| Western and IP cell lysate | **P0013** | Beyotime Biotechnology |
| BCA Protein Quantification Kit | B6169 | UElandy |
| FastPAGE Protein Gels | TSP024 | Beijing Tsingke Biotech Co., Ltd. |
| Colored pre-stained protein markers | TSP021 | Beijing Tsingke Biotech Co., Ltd. |
| BeyoColor™ Prestained Protein Molecular Weight Ladder (6.5-270kD) | P0071 | Beyotime Biotechnology |
| SDS-PAGE protein loading buffer (5X) | BL502B | Biosharp |
| 10×RealBLot Rapid Transfer Buffer | BL609A | Biosharp |
| PVDF membrane | IPVH00010 | MerckMillipore |
| ECL chemiluminescent substrate | BL520B | Biosharp |
| skimmed milk powder | BS102 | Biosharp |
| TBST buffer | BL315B | Biosharp |
| Calcium Ion (Ca^2+^) Concentration Detection Kit | S1063S | Beyotime Biotech Inc |
| ROS Assay Kit | S0034S | Beyotime Biotech Inc |
| Cell Counting Kit-8 | C0038 | Beyotime Biotech Inc |
| Fluo-4 Calcium Assay Kit | S1061S | Beyotime Biotech Inc |
| Revert Aid First Strand cDNA Synthesis Kit | K1622 | Thermo Fisher Scientific |
| 3,3′-diaminobenzidine kit (DAB) | SW01278 | Shenzhen Sunview Technology Co., Ltd. |
| PBS buffer | SW01201 | Shenzhen Sunview Technology Co., Ltd. |
| 2X Universal SYBR Green Fast qPCR Mix | RK21203 | Abclonal |
| ABScript III RT Master Mix for qPCR | RK20428 | Abclonal |
| Normal goat serum | C0265 | Beyotime Biotech Inc |
| Dulbecco's Modified Eagle Medium (No Glucose) | 11966025 | Gibco |
| Dulbecco's Modified Eagle Medium (High Glucose) | 21013024 | Gibco |
| Penicillin Streptomycin | 15140122 | Gibco |
| Fetal Bovine Serum | 10099141c | Gibco |
| 0.25% Trypsin-EDTA (1X) | 25200056 | Gibco |
| Hematoxylin stain | SW01892 | Shenzhen Sunview Technology Co., Ltd. |
| Hematoxylin differentiation solution | SW02563 | Shenzhen Sunview Technology Co., Ltd. |
| Hematoxylin bluing solution | SW01356 | Shenzhen Sunview Technology Co., Ltd. |
| EDTA antigen retrieval solution (pH 9.0) | G1203 | Servicebio |
| EDTA antigen retrieval solution (pH 8.0) | G1206 | Servicebio |
